# Recent Additions to the Medical Talent of Atlanta

**Published:** 1879-11-20

**Authors:** 


### *RECENT ADDITIONS TO THE MEDICAL TALENT OF ATLANTA.*

Of these we mention *Prof. A. S. Payne, of Virginia*, recently elected President of the Virginia Medical Society, which honor he declined to accept the Chair of Practice in the *Southern Medical College.*

Prof. E. J. Hallock, of New York, appointed to the Chair of Chemistry *in the same school.*

Prof. Wm. Perrin Nicolson, from Virginia, appointed to the Chair of Anatomy, and Dr. Lindsay Johnson, of Northern Georgia, to the place of *Demonstrator of Anatomy*, etc., in the same school. These are gentlemen of eminent ability, whose talent will redound to the benefit of the fortunate students of this new and rising Institution.

